# Spectrophotometric analysis of stability of gold nanoparticles during catalytic reduction of 4-nitrophenol

**DOI:** 10.3906/kim-2004-52

**Published:** 2021-02-17

**Authors:** Farhat SAIRA, Samia SALEEMI, Humaira RAZZAQ, Rumana QURESHI

**Affiliations:** 1 Nanoscience and Technical Division, National Centre for Physics (NCP), Islamabad Pakistan; 2 Department of Chemistry, Quaid-i-Azam University, Islamabad Pakistan; 3 Department of Chemistry, University of Wah, Rawalpindi Pakistan

**Keywords:** Spectrophotometry, gold nanoparticles, surface plasmon resonance, optical density, rate constant

## Abstract

Spectrophotometric monitoring of 4-nitrophenol (4-NP) reduction by sodium borohydride (NaBH_4_) using gold nanoparticles (GNPs) as a catalyst has been extensively studied, but the stability of GNPs in terms of change in the surface plasmon resonance (SPR) at different temperatures has not been explored. In the present investigation, our aim was to evaluate the SPR stability of GNPs as a catalyst during the reduction of 4-NP at different elevated temperatures (i.e. 30–60 °C) and sodium borohydride concentrations. Sensitivity of this degradation process toward concentration of GNPs at a range of temperatures is also evaluated. The spectrophotometric results reveal that up to 45 °C, 12 ± 1.5 nm catalyst has a consistent optical density (OD) during the entire 4-NP reduction process, which is related to the surface integrity of catalyst atoms. As the temperature approached 50 °C, the OD gradually decreased and showed a blue shift as the reaction proceeded, which could be related to a decrease in particle size or surface dissolution of gold atoms. The present study may find application in the design of catalysts for the reduction of organic pollutants in industrial wastewater at a range of temperatures.

## 1. Introduction

Pal et al. were the first to identify catalyst-mediated conversion of 4-nitrophenol (4-NP) to 4-aminophenol (4-AP) [1]. The product of this reaction is 4-AP, an effective intermediate in the synthesis of painkillers. There are other uses for it in the photographic and cosmetic industries. Thermodynamics predict the feasibility of this process, but kinetically this reaction is not favorable, i.e. it does not occur in the absence of a catalyst if left for 2 days [2]. This reduction reaction is feasible in the presence of a nanocatalyst. Reaction monitoring is usually carried out by UV-Vis spectroscopy at a 400-nm wavelength. Spectrophotometry is a method applied to assess the quantitative level of an analyte in a particular solution. The basic principle is the absorption of light at a particular wavelength as it passes through the solution. This information is good enough to calculate different kinetic parameters such as entropy of activation, rate constant, activation energies, and frequency factor [3–5]. Organic pollutants are removed by various techniques from industrial wastes, including photocatalysis, membrane filtration, chemical precipitation, physical adsorption, and catalytic degradation. The present degradation method to remove organic pollutant is a simple, efficient, and applicable technique which is an alternative green method, because it has low energy consumption and does not produce a large amount of metal oxide sludge [6].

Gold nanoparticles (GNPs) possess fascinating optical, chemical, and electronic properties; because of these properties, they are used in the fields of catalysis, electronics, and biomedicine. In the field of catalysis, much higher rates than bulk metal have been reported owing to the unique properties of nanocrystals in catalyzing different reaction types [7–14]. Unsaturated valencies of surface atoms render nanocrystals with relatively higher surface energies. Size, shape, concentration, and temperature have been the variables focused on through the years for catalytic testing of nanomaterials [15–39]. However, the stability of the catalyst during the course of a catalytic reaction has not been reported in detail at different ranges of temperatures and concentrations of borohydride (BH). Previously, the SPR stability of gold nanoparticles has been evaluated for the reduction of potassium hexacyanoferrate (III) at room temperature only; it was observed that SPR remained stable over the course of the reduction reaction [20]. This fact is reported in detail in the Discussion section. Conversion of 4-NP and potassium ferricyanide by SBH is used as a model reaction [17–19].

## 2. Materials and methods

**Figure 1:**
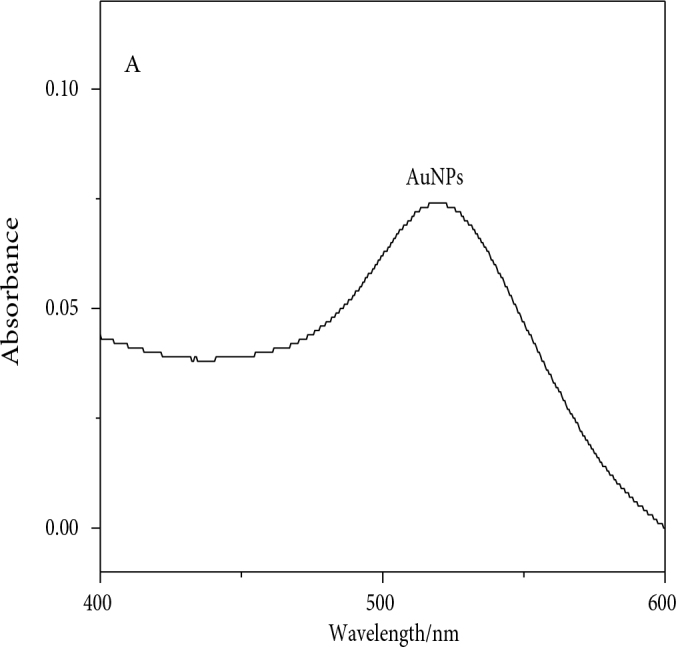

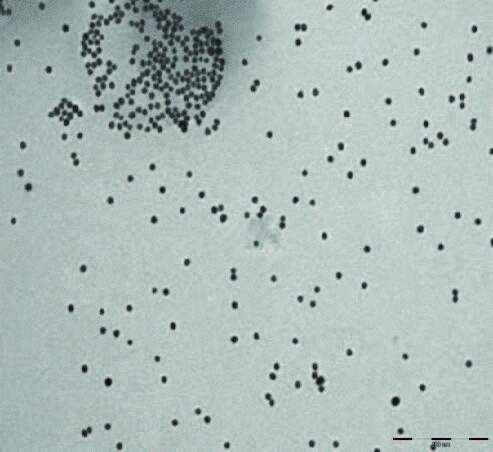
UV-visible spectrum (A) and TEM image (B) of gold nanoparticles.

All of the required chemicals for the present study were of analytical grade and were used as received. A previously reported method was used to synthesize GNPs (gold nanoparticles) of 12 ± 1.5 nm diameter [40]. Briefly, trisodium citrate (2 mL of 1% solution) was added dropwise to a boiling 20-mL (1 mM) gold salt solution. The solution was stirred until a red color was obtained, and stored in a cold location for further use. A Shimadzu 1601 (Shimadzu, Kyoto, Japan) was used to measure spectroscopic data; TEM at 100 kV (FEI Tecnai Spirit; FEI, Hillsboro, OR, USA) was used to characterize GNPs (Figure 1). Ultrasonication was carried out for 2 min before each catalytic experiment with these GNPs.

The reaction mixture consisted of 100 µL each of 8nM GNPs and 1 mM 4-NP added to a standard quartz cuvette. The volume was brought up to 3.5 mL with water. Freshly made and chilled NaBH_4_ (100 µL, 100mM) was added to the cuvette. The pale yellow color of the 4-nitrophenol changed immediately to dull green. Spectrophotometric changes were recorded in a time span of 1–3 min according to the reaction conditions. The rate constant was determined depending on experimental factors. An extinction coefficient of 12 ± 1.5 nm NPs was calculated from the UV-Vis spectrum using the method given in the literature [41], and was found to be 2 × 10^8^ M^-1^ cm^-1^.

## 3. Results and discussion

GNPs were synthesized using the Frens method [40] and were utilized for catalytic reduction of 4-NP. Spectrophotometric monitoring of stability of GNPs during the course of the reaction was carried out. The stability of GNPs was studied in terms of surface plasmon resonance (SPR), as it is highly sensitive to the chemical environment, i.e *.* the refractive index of the species surrounding GNPs. SPR is a phenomenon that occurs as a result of the cooperative oscillation of valence electrons in the metallic nanoparticles. This research article deals with this physical phenomenon in GNPs during catalytic reduction reaction.

### 3.1. Stability of GNPs at different temperatures

**Figure 2:**
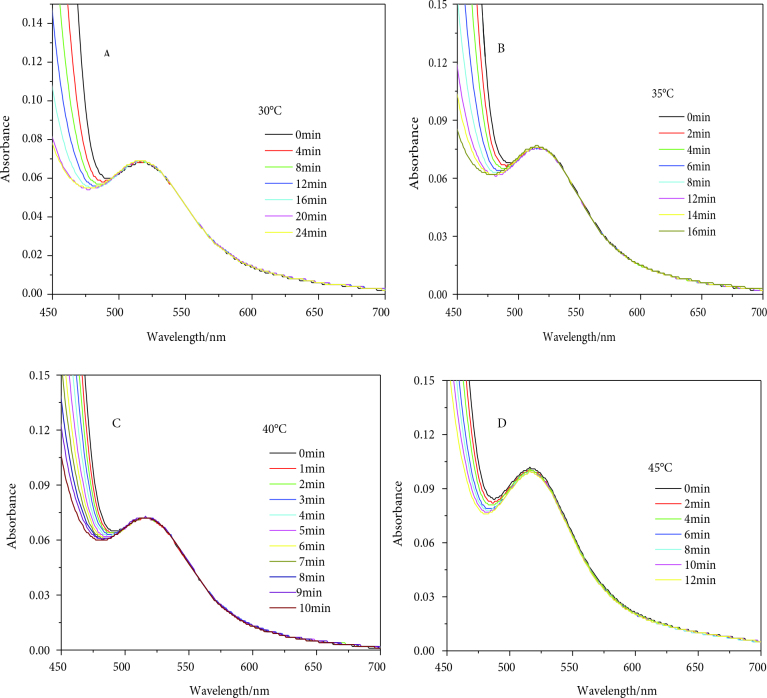
Stability of GNPs in terms of their consistent OD at 30 °C (A), 35 °C (B), 40 °C (C), and 45 °C.

**Figure 3:**
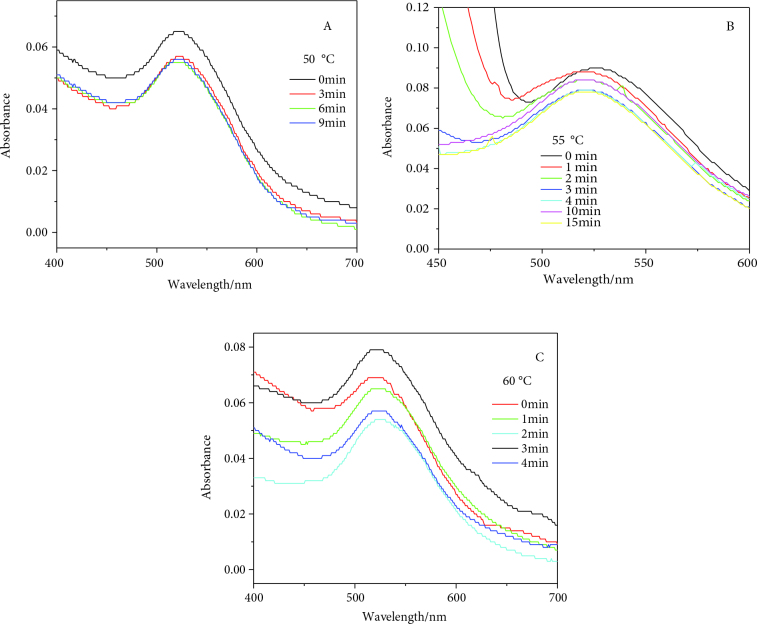
GNPs dissolution at higher temperatures 50 °C (A), 55 °C (B), and 60 °C (C) during the course of reduction of 4-NP.

The stability of GNPs at different temperatures was a new finding for this article. GNPs of 12 ± 1.5 nm diameter were found to be fairly stable at 30, 35, 40, and 45 °C for the reduction of 4-NP (Figure 2). At the studied range of temperatures, the GNPs were capable of withstanding the constant collisions of reactants and solvent molecules. Additionally, the surface atoms of the GNPs were not dissolved during the course of the reaction, as observed by its consistent OD. However, as the temperature increased from 45 to 50 °C, signs of catalyst dissolution started to appear, as observed from the gradual decrease in the OD values (Figure 3). This can be inferred from the collisions of reactant as well as solvent molecules against the catalyst surface as a result of their increased kinetic energy at higher temperatures, which leads to the surface atoms’ dissolution into the reaction mixture. This process of dissolution continued as the temperature varied from 50 to 60 °C, but no sign of aggregation was observed (see Figures 2 and 3).

**Figure 4:**
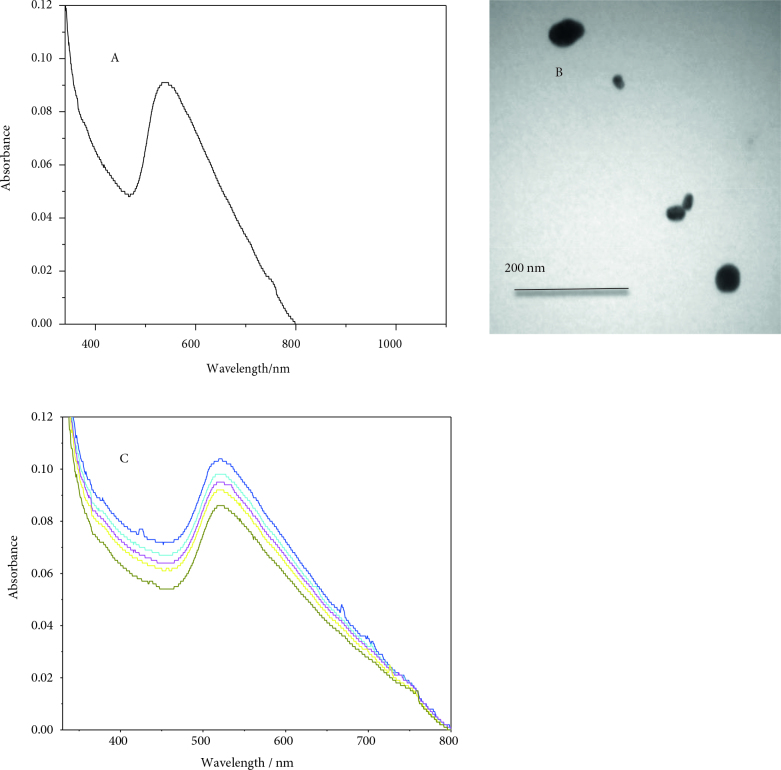
UV-visible spectrum (A), TEM image (B) of 30 ± 5 nm gold nanoparticles, and GNPs (30 nm diameter) dissolution at 50 °C during the course of degradation of 4-NP (C).

Furthermore, the effect of size on the stability of GNPs was examined. For this purpose, 30-nm Au nanoparticles were synthesized and characterized by UV-Vis spectroscopy and TEM as indicated in Figures 4A and 4B. SPR changes for the larger nanoparticles (30 nm) during the reduction of 4-nitrophenol were recorded in the UV-Vis spectra (Figure 4C), and the following conclusion was drawn. The SPR decreased in this case as well, which can be attributed to the dissolution of the surface gold atoms; this is consistent with our previous findings with 12-nm AuNPs. These results for 30-nm AuNPs are different from the results observed for 12-nm AuNPs.

### 3.2. Sensitivity of 4-NP reduction toward GNP concentration

In order to evaluate the sensitivity of this reduction process to GNP concentration, the process was varied from 11.8 nM to 15.5 nM at various temperatures. The kinetic analysis of both of the concentrations was carried out using pseudo-first order kinetics.

(1)lnA=-kt

Plotting lnA vs. time, rate constant values were attained [19,20] as shown in Table,

**Table:** Comparison of kinetic parameters of 4-Nitrophenol reduction reaction at two concentrations of gold nanoparticles.

GNPs conc.(nM)	Rate constant at35 °C (min^-1^)	Rate constant at40 °C (min^-1^)	Rate constant at 45 °C (min^-1^)	Activation energy(kcal/mol)	Frequency factor(min^-1^)	Entropy of activation
11.8	0.019 ± 1.2×10^-3^	0.04 ± 9.6×10^-4^	0.09 ± 2.3×10^-3^	30.24 ± 1.025	3.49 × 10^19^	64.11
15.5	0.099 ± 9.9×10^-4^	0.198 ± 1.1×10^-2^	0.29 ± 8.3×10^-3^	19.87 ± 3.417	1.03 × 10^14^	89.4

where A is depicting absorbance of 4-NP, t is time for reduction, and k is obtained rate constant. It was found that the rate constant of 15.5 nM GNPs was 5.2 times higher than for lower concentrations at 35 °C, 4.9 times higher at 40 °C, and 3.2 times higher at 45 °C as shown in Figure 5. 

**Figure 5:**
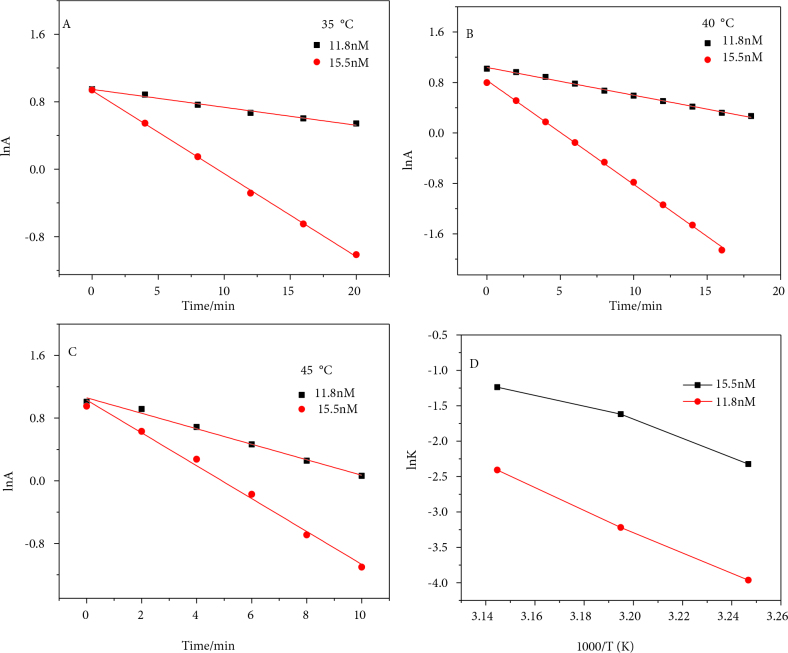
Concentration sensitivity of 4-NP reduction process towards GNPs investigated at different temperatures.

This shows that the extent of the difference in the rate constants gradually decreased as the temperature increased. This may be attributed to the increase in the kinetic energies of the reactants with temperature, which ends in the proximity of both reaction rates. The Arrhenius equation was applied to attain activation energy.

(2)Lnk=lnA-Ea/RT

Here, lnk (rate constant) was plotted against 1/T (temperature in K); the frequency factor k_0_ was obtained from the intercept, and E_a_ was obtained from the slope of the straight line. E_a_ was decreased from 30 to 19 kcal/mol for this small increase in catalyst concentration. On the other hand, entropy values were found to increase from 64 to 89 Cal/mol.K. At 50, 55, and 60 °C, the catalyzed reaction was quick enough for the present ratio (1/637000/3076000 catalyst/4NP/BH) to evaluate the kinetic parameters. No induction period t_0_ was observed for either of the concentrations of the catalysts, and the reaction began immediately after mixing. With the addition of borohydride, a maximum of 9 nm blue shift was observed after mixing the reactants and the GNPs. This blue shift was not found to be linearly dependent upon BH concentration, and there was a saturation limit after which no further shift was observed. In addition, the SPR of GNPs was not seen to be affected by the 4-NP concentration. This effect and stability were monitored with a UV-Vis spectrophotometer at 30, 35, 40, 45, 50, 55, and 60 °C. It was observed that the concentration of the catalyst plays a vital role in terms of kinetic parameters of the reduction reaction. At slightly higher concentrations of the catalyst, the rate of reduction as well as entropy values increase, while activation energy decreases. However, as the temperature increases, the concentration effect becomes negligible and rate constants for both of the concentrations come into proximity. This could be interpreted as dissolution of the surface gold atoms from GNPs as a result of increase in the kinetic energy of the reactants and solvent molecules and their collisions against the superficial atoms of GNPs, which results in decrease of GNPs size.

### 3.3. Effect of BH concentration towards stability and reactivity of GNPs

**Figure 6:**
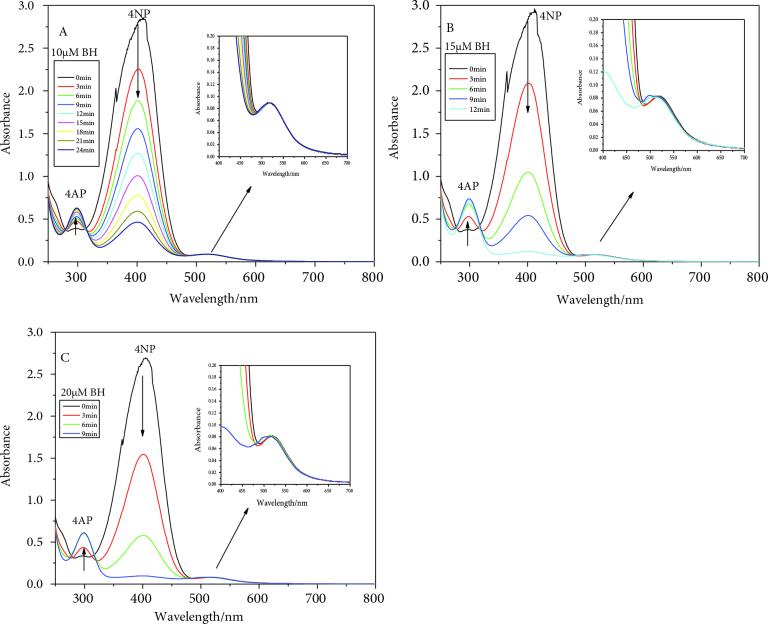
SPR stability of GNPs during catalytic reduction of 4-NP at different sodium borohydride concentrations: 10 μM (A), 15 μM (B), and 20 μM (C). A clear blue shift is seen in the cases of 15 and 20 μM concentrations.

This reduction reaction does not strictly follow pseudofirst-order kinetics, which is why the initial concentrations of 4-nitrophenol, borohydride, and catalyst affect the rate of reaction [37]. The effect of BH concentrations on the stability as well as reactivity of gold nanoparticles during 4-NP reduction was investigated for a range of BH concentrations at room temperature. GNPs were found to be stable (in terms of their consistent optical density) throughout the BH concentration range studied. The dependence of reaction rate on BH concentration is not a linear relationship but a complex one [17,19]. The present studied range shows a linear dependency, although the extent of blue shift was enhanced at the higher concentrations. The higher the hydrid ions surrounding the GNPs, the more displacement of citrate ions will occur [42]; because of this displacement, the SPR shifts toward the lower wavelength region. The extent of the blue shift is because of the change in the refractive index of the surrounding environment of these catalysts, as shown in Figure 6.

The BH adsorbs on the nanoparticle surface and produces H2 gas and hydrid ion; these lighter species with a refractive index of 1 displace water, which has a refractive index of 1.33. Thus, this particular blue shift is not because of the size, shape, or interparticle distance change for the catalysts, but only because of the refractive index change of their surrounding medium [22,23]. Hence, there was only one abrupt SPR blue shift for these nanocatalysts even after the completion of the whole 4-NP reduction, unlike the gold nanocages, where a time-dependent gradual blue shift was observed because of the inner and outer surfaces of the cavities being available to the reactants [22,23]. The present ratio of GNPs/4NP/BH did not contribute to the aggregation of the GNPs, as reported previously.

**Figure 7:**
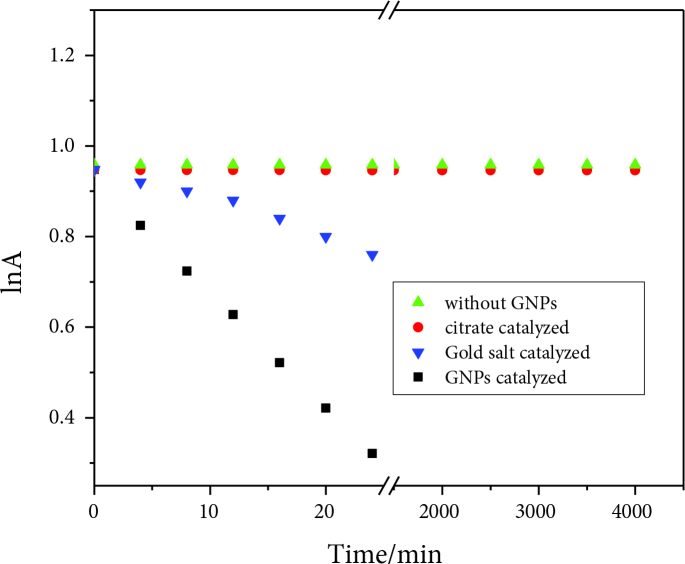
Control experiment for 4-NP reduction with borohydride in the presence and absence of GNPs at room temperature.

BH is known to inject electrons on the exterior surface of gold nanoparticles, which causes the removal of the citrate layer from their surface. If this displacement is effective enough to cause anisotropy of charges on the GNPs’ surface, it can lead to the aggregation of the catalyst [15,36]. It has been reported that capping material is usually stripped off during the synthesis or catalytic reaction; this may contribute to the nanocatalyst aggregation [15 **]** , which was not observed in the present case, with varying concentrations from10 µM to 20 µM. The Langmuir–Hinshelwood model was applied to interpret this reduction reaction [18,19,21]. According to this model, both reactants must be adsorbed to the exterior of the nanocatalyst to react. This reaction was found to be controlled by kinetics, where the transportation of the reactants through the solution was not the rate-determining step; rather, the formation of 4-AP was the rate-determining step. The adsorbed reactants may react, and the product is desorbed from the exterior of GNPs. In a control experiment, there was no reduction of 4-NP observed in the absence of GNPs or in the presence of trisodium citrate, but gold salt solution was found to degrade 4-NP to some extent. The plausible reason for this effect of gold salt solution was the formation of larger-sized GNPs inside the reaction mixture, which catalyzed this reaction to some extent as depicted in Figure 7.

## 4. Conclusion

The stability of any catalyst is important during the course of catalytic reaction. We have studied surface plasmon resonance (SPR) stability of colloidal gold nanoparticles during the catalytic reduction of 4-NP (an organic pollutant) at different temperature conditions and BH concentrations. The SPR shift of GNPs or the formation of 4-nitrophenolate ion can be taken as evidence for adsorption of both reactants on the surface of GNPs, proving the Langmuir–Hinshelwood model. The concentration of GNPs varied from 11.8 nM to 15.5 nM at various temperatures. It was found that the rate constant of 15.5 nM GNPs was 5.2 times higher than for the lower concentration at 35 °C; the difference decreased with increasing temperature. This study may find applications in spectrophotometrically designing catalysts for industrial wastewater removal.

## Conflict of interest

The authors declare no conflict of interest regarding the publication of this work.
